# Development of a Radiomics-Based Model to Predict Graft Fibrosis in Liver Transplant Recipients: A Pilot Study

**DOI:** 10.3389/ti.2023.11149

**Published:** 2023-09-01

**Authors:** Fakhar Ali Qazi Arisar, Emmanuel Salinas-Miranda, Hamideh Ale Ali, Katherine Lajkosz, Catherine Chen, Amirhossein Azhie, Gerard M. Healy, Dominik Deniffel, Masoom A. Haider, Mamatha Bhat

**Affiliations:** ^1^ Ajmera Transplant Program, Toronto General Hospital, University Health Network, Toronto, ON, Canada; ^2^ Division of Gastroenterology & Hepatology, Department of Medicine, University of Toronto, Toronto, ON, Canada; ^3^ National Institute of Liver and GI Diseases, Dow University of Health Sciences, Karachi, Pakistan; ^4^ Lunenfeld Tanenbaum Research Institute, Sinai Health System, Mount Sinai Hospital, Joseph and Wolf Lebovic Health Complex, Toronto, ON, Canada; ^5^ Joint Department of Medical Imaging, University Health Network/Sinai Health System, Toronto, ON, Canada; ^6^ Department of Biostatistics, Princess Margaret Cancer Centre, University Health Network, Toronto, ON, Canada; ^7^ Toronto General Hospital Research Institute and Institute of Medical Sciences, University of Toronto, Toronto, ON, Canada

**Keywords:** CT scan, imaging biomarkers, machine learning, artificial intelligence, prognostic model

## Abstract

Liver Transplantation is complicated by recurrent fibrosis in 40% of recipients. We evaluated the ability of clinical and radiomic features to flag patients at risk of developing future graft fibrosis. CT scans of 254 patients at 3–6 months post-liver transplant were retrospectively analyzed. Volumetric radiomic features were extracted from the portal phase using an Artificial Intelligence-based tool (PyRadiomics). The primary endpoint was clinically significant (≥F2) graft fibrosis. A 10-fold cross-validated LASSO model using clinical and radiomic features was developed. In total, 75 patients (29.5%) developed ≥F2 fibrosis by a median of 19 (4.3–121.8) months. The maximum liver attenuation at the venous phase (a radiomic feature reflecting venous perfusion), primary etiology, donor/recipient age, recurrence of disease, brain-dead donor, tacrolimus use at 3 months, and APRI score at 3 months were predictive of ≥F2 fibrosis. The combination of radiomics and the clinical features increased the AUC to 0.811 from 0.793 for the clinical-only model (*p* = 0.008) and from 0.664 for the radiomics-only model (*p* < 0.001) to predict future ≥F2 fibrosis. This pilot study exploring the role of radiomics demonstrates that the addition of radiomic features in a clinical model increased the model’s performance. Further studies are required to investigate the generalizability of this experimental tool.

## Graphical Abstract

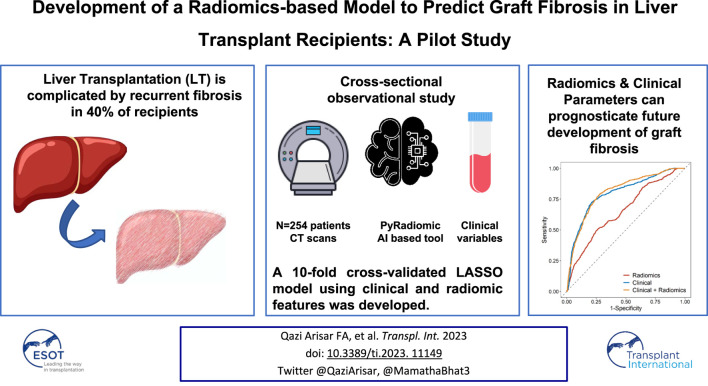

## Introduction

Short-term survival rates after liver transplant (LT) have continued to improve over time, with advances in immunosuppression and post-transplant care [1]. However, this has not been matched by gains in long-term survival rates [1–3]. Recurrent fibrosis following LT continues to be a significant factor impacting long-term graft and patient survival. Advanced graft fibrosis occurs in approximately 37%–43% of LT recipients [4, 5]. Development of Stage 2 graft fibrosis within the first-year post-transplant is associated with reduced graft and patient survival [6, 7].

Graft fibrosis may occur due to repeated episodes of rejection, recurrence of primary disease, or recurrent and *de novo* non-alcoholic steatohepatitis (NASH) [8]. Liver enzymes give unreliable information to assess progressive graft fibrosis over time when preventive interventions are possible. Furthermore, repeated liver biopsies for screening and monitoring in LT patients are not practically feasible given the potential risks associated with an invasive procedure and expense [9, 10]. Longitudinal serum biomarkers and transient elastography are helpful in identifying patients who have developed advanced liver fibrosis [4, 5, 11]. However, more robust non-invasive tools are required to identify those at the highest risk of developing advanced graft fibrosis in the long term.

**Radiomics** is a method of converting medical images into high-dimensional, mineable quantitative data, followed by subsequent data analysis for decision support [12]. Radiomics has been used successfully to assess liver fibrosis on CT images in chronic liver disease [13, 14], while for LT patients it has been mainly focused on predicting early recurrence of hepatocellular carcinoma (HCC) post-transplant using pretransplant CT images [15, 16]. To our knowledge, there have been no studies to date that explore the utility of radiomic features on post-transplant images in predicting graft fibrosis in solid organ transplant recipients.

**FIGURE 1:**
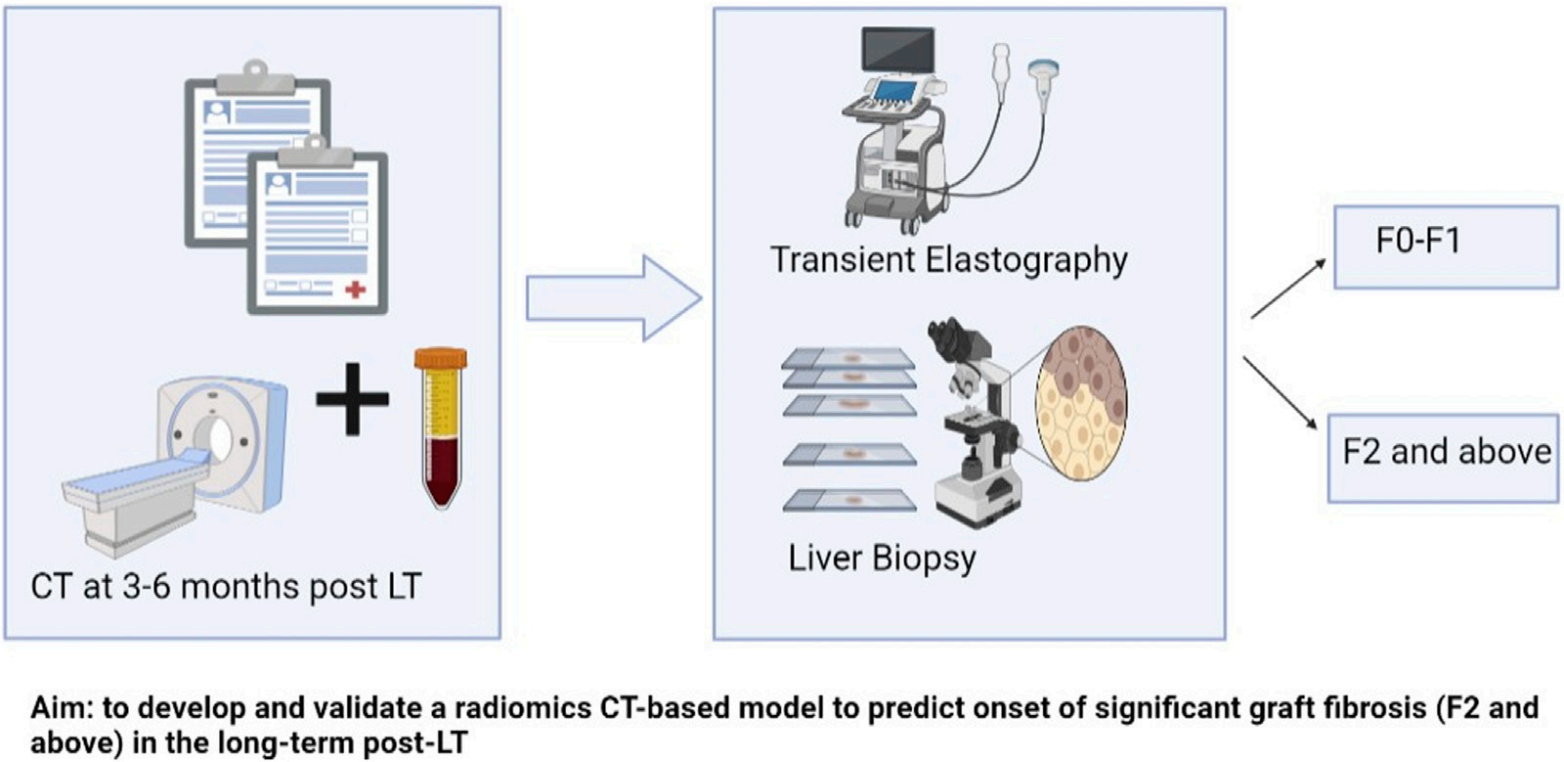
Schematic presentation of aim and methods.

In this study, we aimed to develop and validate a radiomics-based model to predict the onset of >F2 graft fibrosis in the long term post-LT. Figure 1 represents the schematic presentation of our aim. We opted for F2 or more fibrosis as it is categorized as clinically significant fibrosis [17]. It is important to identify patients at risk of clinically significant fibrosis in the long term. Earlier identification of such higher-risk patients will enable the implementation of preventive measures that could save the graft. We hypothesized that radiomic features such as subtle perfusion, and biliary and parenchymal changes early post-LT could provide insight into the long-term life span of the graft, beyond the longitudinal clinical and laboratory information available.

## Materials and Methods

### Patient Population

**FIGURE 2:**
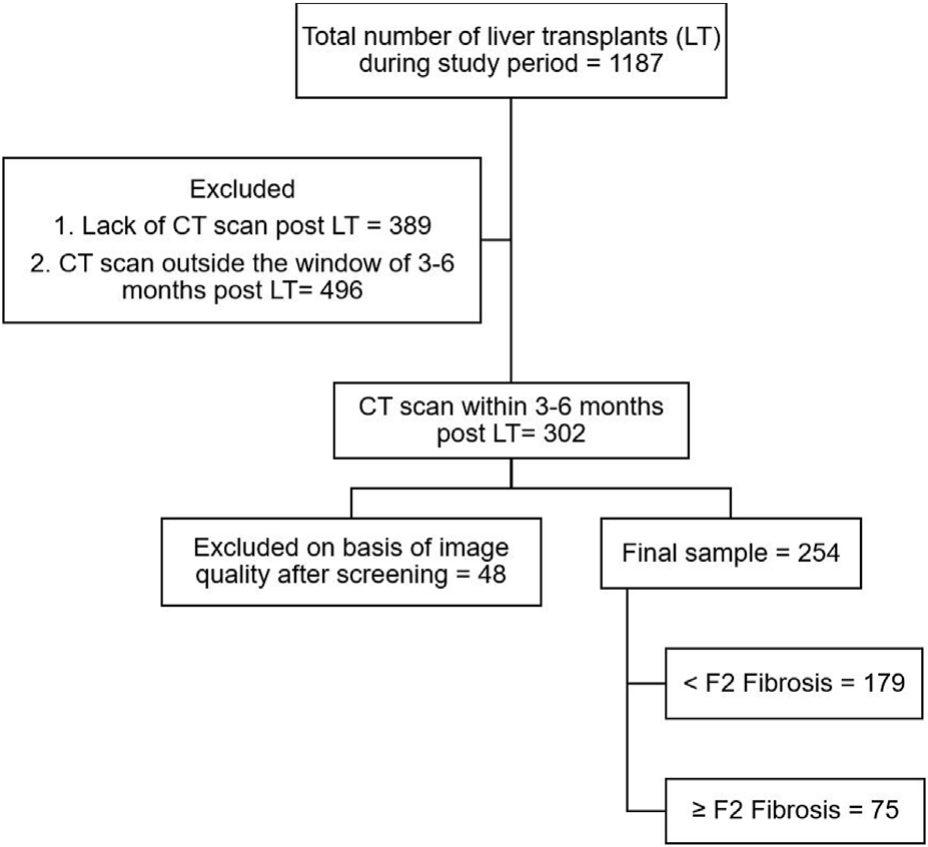
Study flowchart.

This retrospective multi-center study was done at University Health Network and Mount Sinai Hospital, Toronto, and included all adult patients who underwent LT between January 2009 and December 2018 and had post-transplant contrast-enhanced computed tomography (CT) scan available, including a venous phase with/without an arterial phase, at 3–6 months after LT. This period for CT scans was selected in order to give time for the post-surgical changes to reverse, which takes a few weeks [18]. Missing clinical characteristics data were multiply imputed ten times using five iterations of multiple imputation by chained equations. The model coefficients and performance measures were pooled using Rubin’s rules. The study flowchart is depicted in Figure 2.

We collected data on demographics (date and type of LT; recipient and donor age; recipient sex, height, weight, and body mass index (BMI); primary indication for LT; comorbidities such as diabetes, hypertension, dyslipidemia, cardiovascular disease, dialysis status, smoking, and alcohol consumption; recurrence of primary etiology (any time post LT); recurrence of hepatocellular carcinoma (HCC) or cholangiocarcinoma; development of fibrosis; re-transplantation; and death post-LT), laboratory tests at various intervals post-transplant (platelets, total bilirubin, AST, ALT, ALP, INR, sodium, creatinine, eGFR, APRI, Fib-4), and the immunosuppression regimen.

The study’s primary endpoint was Fibrosis stage F2 or greater (≥F2) quantified by either transient elastography (TE) or liver biopsy. Liver biopsy was indicated either as a prerequisite of hepatitis C treatment in the interferon era or on a need basis such as for elevated liver enzymes. Since the availability of TE (2018), all patients at our center underwent routine TE annually. TE was not available for many patients due to the wide range of the study period; hence we used both TE and liver biopsy whichever was available, given their comparable performance in staging liver fibrosis, even in post-liver transplant patients [5, 19, 20]. The protocol was approved by our institutional Research Ethics Board (REB # CAPCR ID: 19-6159).

Liver biopsy samples were considered adequate if they were at least 15 mm long and carried at least 6 complete portal tracts, and were read by an expert liver pathologist [21]. Fibrosis stages in biopsy samples were scaled based on the METAVIR score, from F0 to F4 (F0: No fibrosis–F1: Portal fibrosis without septa–F2- Portal fibrosis with few septa–F3: fibrosis with numerous bridging septa–and F4: cirrhosis) [22].

Transient elastography was done using the Fibroscan device (Echosense, Paris) with standard M or XL (for obese patients, as guided by the device) probes. Liver stiffness measurement (LSM) expressed in kilopascals (kPa) identified graft fibrosis severity. LSM ≥7.4 was considered significant graft fibrosis (F2 and above) based on the results of a recent prospective study that showed a sensitivity of 0.9 for this cutoff in LT recipients with different underlying pathologies. Only examinations with at least 10 measurements and a successful rate >60%, with an interquartile range <30% of the median value were considered reliable for the study [23].

### CT Feature Extraction

One radiologist (ES) manually contoured a 30 mm diameter spherical volume of interest (VOI) in the posterior aspect of the right liver lobe (segment V or VI) in the arterial and portal phase of each patient. The portal branches and hepatic veins were excluded from segmentation. A radiologist with more than 20 years of experience in abdominal radiology (MH) confirmed the contours. 3D Slicer v4.11.2 ^1^, an open segmentation software was used. Feature extraction was performed with PyRadiomics version 3.0, an image biomarker standardization initiative compliant analytic library [24]. CT images with the region of interest in the right liver lobe are depicted as a Supplementary Figure S1. Typical CT parameters and hyperparameters used for analysis are listed in Supplementary Tables S1–S3. In total, 116 non-filtered features were extracted.

### Statistical Analysis

Baseline variables were compared between cohorts using the Mann-Whitney U test and Fisher’s Exact test for continuous and categorical variables, respectively. The association of the clinical variables and the radiomic features with ≥F2 was assessed by using univariable and multivariable generalized logistic regression models. Clinical features with a skewed distribution were log transformed.

Three models, radiomics only, clinical only, and radiomics + clinical, were developed to predict ≥ F2 on the liver graft. Radiomic features were standardized using Z-transformation and features with zero variance were removed. Following this, radiomic features that were significant (*p* < 0.05) in the fitted univariable logistic regression models were retained. These features were introduced in the Least Absolute Shrinkage and Selection Operator (LASSO) to generate the final radiomic model and were validated using 10-fold cross-validation. The clinical-only model was developed using a similar methodology. All the clinical features that were statistically significant (*p* < 0.05) in the univariable model were retained and then incorporated into a 10-fold cross-validated LASSO model to generate a final list of clinical features. The clinical and radiomics model included all features from the clinical-only and radiomics-only models. All models were internally validated using 10-fold cross-validation repeated 10 times. At the end, model performance was tested on patients with liver biopsy-determined fibrosis by excluding patients with fibroscan-determined fibrosis.

The mean area (AUC) under the receiver operator characteristic curve (ROC) was used to assess the discrimination of the radiomics and the clinical models. 95% confidence intervals (CI) were calculated based on 1,000 bootstrap replicates. Model calibration was visually assessed using calibration curves and quantified using average absolute calibration error. The mean ROC curve was plotted for each model. DeLong’s test was used to formally compare differences in AUCs across models. Time to ≥F2 fibrosis was estimated using cumulative incidence functions; death without fibrosis was considered a competing risk. Patients who did not die or develop fibrosis were censored at the date of the last follow-up. Cumulative incidence function curves were stratified by radiomic features and differences in curves were evaluated using Gray’s test.

To assess confounding between each selected clinical characteristic and the selected radiomics features when predicting ≥F2 fibrosis, separate multivariable logistic regression models incorporating each feature and the selected radiomics features were fit. A difference of 10% between the univariable and adjusted odds ratio was considered to be indicative of confounding.

All statistical tests were two-tailed, and *p* < 0.05 was considered statistically significant. Statistics were performed using R v4.0.0 (R project for statistical computing) [25]. Methods and results were reported according to the Transparent Reporting of Multivariable Prediction Model for Individual Prognosis or Diagnosis (TRIPOD) statement [26].

## Results

Out of 1,188 patients who underwent liver transplants during the study period, a total of 254 patients met the inclusion criteria, specifically due to the need for CT scans at 3–6 months post-LT. Patients were mostly male (76%), with a mean age of 56.3 ± 10.2 years at transplant. The most common etiology of the underlying liver disease was viral (54%). Of those included, 204 (80.3%) patients had HCC and/or cholangiocarcinoma before transplant and 75% of patients underwent deceased donor liver transplants. The median duration of follow-up was 6.7 (1.1–12.4) years. Table 1 summarizes the demographic and laboratory variables.

**TABLE 1:** Demographic and clinicopathological characteristics.

Variable		Full sample (*n* = 254)	<F2 fibrosis (*n* = 179)	≥F2 fibrosis (*n* = 75)	*p*-value*
Primary diagnosis	n (%)				0.74
Viral		136 (54)	91 (51)	45 (60)	
Alcohol		38 (15)	29 (16)	9 (12)	
Autoimmune liver diseases		31 (12)	22 (12)	9 (12)	
NASH		22 (9)	16 (9)	6 (8)	
Other		27 (11)	21 (12)	6 (8)	
Liver malignancy pre-LT	n (%)				0.59
Cholangiocarcinoma		3 (1)	3 (1.7)	0 (0)	
HCC		196 (77)	140 (78.2)	56 (74.7)	
HCC + Cholangiocarcinoma		4 (1.5)	4 (2.2)	0 (0)	
HCC + Gall bladder carcinoma		1 (0.4)	1 (0.6)	0 (0)	
None		50 (19.6)	31 (17.3)	19 (25.3)	
Transplant Type	n (%)				**0.0079**
Deceased cardiac donor		26 (10)	13 (7)	13 (17)	
Living donor		62 (25)	39 (22)	23 (31)	
Deceased brain-dead donor		164 (65)	125 (71)	39 (52)	
Age at transplant (years)	Mean (SD)	56.3 (10.2)	57.2 (10.2)	54.0 (9.8)	**<0.001**
Sex	n (%)				0.87
Female		59 (23)	41 (23)	18 (24)	
Male		195 (77)	138 (77)	57 (76)	
BMI (Kg/m^2^)	Mean (SD)	27.1 (5.1)	27.2 (5.0)	27.0 (5.3)	0.66
BMI Category	n (%)				0.43
<30		186 (74)	133 (76)	53 (71)	
≥30		65 (26)	43 (24)	22 (29)	
Missing		3	3	0	
Donor Age (Years)	Mean (SD)	44.5 (16.5)	44.0 (17.1)	45.6 (15.2)	0.40
	Missing	2	2	0	
Diabetes Pre LT	n (%)	80 (31)	58 (32)	22 (29)	0.66
Hypertension pre-LT	n (%)	85 (33)	63 (35)	22 (29)	0.39
Dyslipidemia pre-LT	n (%)	33 (13)	26 (15)	7 (9)	0.51
Cardiovascular disease pre-LT	n (%)	20 (8)	15 (8)	5 (7)	0.80
Smoking pre-LT	n (%)	136 (54)	98 (55)	38 (51)	0.58
Dialysis pre-LT	n (%)	2 (1)	0 (0)	2 (3)	0.086
Diabetes post-LT	n (%)	125 (49)	93 (52)	32 (43)	0.16
Hypertension post-LT	n (%)	155 (61)	104 (58)	51 (68)	0.16
Dyslipidemia post-LT	n (%)	69 (27)	49 (28)	20 (27)	1
Cardiovascular disease post-LT	n (%)	33 (13)	21 (12)	12 (16)	0.41
Dialysis post-LT	n (%)	29 (11)	19 (11)	10 (13)	0.52
Smoking post-LT	n (%)	20 (8)	17 (9)	3 (4)	0.2
Alcohol consumption post-LT	n (%)	9 (4)	6 (3)	3 (4)	0.73
HCC/Cholangiocarcinoma Recurrence	n (%)	41 (16)	28 (16)	13 (17)	0.71
Recurrence of the Primary diagnosis	n (%)	93 (37)	43 (24)	50 (67)	**<0.001**
Platelet at Transplant (x10^9^/L)	Median (Min, Max)	164 (29, 782)	169 (38, 782)	158 (29, 584)	0.43
Platelets at 3 months (x10^9^/L)	Median (Min, Max)	157 (15, 532)	162 (39, 532)	148.5 (15, 446)	0.044
AST at Transplant	Median (Min, Max)	1040.5 (96.0, 10300.0)	1,006 (96, 8,209)	1,155 (144, 10,300)	0.48
AST at 3 months (IU/L)	Median (Min, Max)	28 (9, 358)	26 (9, 358)	42 (14, 268)	**<0.001**
	Missing	1	1	0	
ALT at Transplant (IU/L)	Median (Min, Max)	747.5 (55.0, 7509.0)	721 (55, 7,509)	770 (128, 5,229)	0.71
ALT at 3 months (IU/L)	Median (Min, Max)	35 (3, 522)	30 (3, 522)	53 (7, 493)	**<0.001**
	Missing	2	2	0	
ALP at Transplant (IU/L)	Median (Min, Max)	103.5 (37.0, 1791.0)	103 (37, 1,791)	105 (44, 1,279)	0.91
ALP 3 months (IU/L)	Median (Min, Max)	118 (39, 2,197)	108 (39, 565)	131 (49, 2,197)	**0.0041**
	Missing	2	2	0	
Total Bilirubin at Transplant (µmol/L)	Median (Min, Max)	60 (6, 613)	58 (6, 613)	65.5 (7.0, 512.0)	0.11
Total Bilirubin 3 months (µmol/L)	Median (Min, Max)	10 (3, 169)	9 (3, 169)	13 (3, 73)	**<0.001**
	Missing	2	1	1	
INR at LT	Median (Min, Max)	1.8 (0.8, 5.2)	1.8 (0.8, 4.0)	1.8 (1.0, 5.2)	0.77
INR 3 months	Median (Min, Max)	1.0 (0.9, 3.0)	1.0 (0.9, 3.0)	1.0 (0.9, 1.9)	0.87
	Missing	5	5	0	
Serum Creatinine at Transplant (µmol/L)	Median (Min, Max)	84 (43, 359)	84 (48, 307)	84 (43, 359)	0.61
Serum Creatinine 3M (µmol/L)	Median (Min, Max)	91 (28, 541)	91.5 (28.0, 159.0)	87 (44, 541)	0.32
	Missing	1	1	0	
Serum Sodium at Transplant (mmol/L)	Mean (SD)	140.3 (4.5)	139.9 (4.3)	141.1 (4.9)	**0.026**
Serum Sodium 3 months (mmol/L)	Mean (SD)	139.9 (3.1)	139.9 (3.3)	139.9 (2.7)	0.69
	Missing	1	1	0	
Immunosuppressant 3 months	n (%)				**<0.001**
Cyclosporine		63 (25)	27 (15)	36 (48)	
Sirolimus		7 (3)	5 (3)	2 (3)	
Tacrolimus		184 (72)	147 (82)	37 (49)	
APRI at 3 months	Median (Min, Max)	0.5 (0.1, 25.0)	0.5 (0.1, 8.7)	0.8 (0.1, 25.0)	**<0.001**
	Missing	7	6	1	
Fib-4 at 3 months	Median (Min, Max)	1.7 (0.2, 37.7)	1.5 (0.2, 11.9)	2.2 (0.2, 37.7)	**<0.001**
	Missing	8	7	1	
Duration of Follow-up (Years)	Median (Min, Max)	6.7 (1.1, 12.4)	6.6 (1.1, 12.4)	7.4 (1.1, 12.1)	0.79

Notes: * Mann-Whitney U test for continuous covariates, and Fisher’s Exact test for categorical covariates.

Abbreviations: ALP, alkaline phosphatase; ALT, alanine transaminase; AST, aspartate transaminase; BMI, body mass index; HCC, hepatocellular carcinoma; INR, international normalized ratio; LT, liver transplant; NASH, non-alcoholic steatohepatitis; SD, standard deviation.

Bold values represents that the *p* value < 0.05.

In total, 75 (29.5%) patients developed ≥F2 fibrosis. The median time from transplant to ≥F2 fibrosis was 19 (4.3–121.8) months, while the time from CT scan was 14.1 (0–116) months. Recurrence of primary etiology was noted in 93 (37%) patients, while 41 (16%) had a recurrence of HCC/cholangiocarcinoma in the long term. Patients who developed ≥ F2 fibrosis in the long term had more deceased cardiac donor (DCD) LTs (17% vs. 7%, *p* = 0.0079), younger age at transplant (54 ± 9.8 vs. 57.2 ± 10.2, *p* < 0.001), higher rate of primary disease recurrence (67% vs. 24%, *p* < 0.001), elevated liver enzymes at 3 months post-LT, and less frequent use of tacrolimus at 3 months post-LT (49% vs. 82% *p* < 0.001) as described in Table 1.

The LASSO algorithm selected two radiomic features, original first-order maximum and original first-order root mean squared. The two were highly correlated with a Spearman correlation coefficient of 0.86, and therefore only the first-order maximum (maximum liver attenuation) was selected for the radiomics model (OR: 0.52 [95% CI: 0.38–0.71], *p* < 0.001). The results from the univariable logistic regression models for all radiomic features are presented in Supplementary Table S4.

### Association of Radiomics-Score and Clinical Variables With Graft-Fibrosis

In the multivariable generalized regression analysis, primary etiology of alcohol (OR 5.49, 95% CI 1.60–18.80, *p* = 0.007), donor age (OR 1.04, 95% CI 1.01–1.07, *p* = 0.002), recipient age at transplant (OR 0.95, 95% CI 0.91–0.98, *p* = 0.004), recurrence of primary etiology (OR 6.31, 95% CI 2.46–16.16, *p* < 0.001), brain-dead donor (OR 0.16, 95% CI 0.05–0.48, *p* = 0.001), tacrolimus use at 3 months post-LT (OR 0.27, 95% CI 0.11–0.65, *p* = 0.004), and APRI score at 3 months post-LT (OR 1.93, 95% CI 1.26–2.95, *p* = 0.003) were the clinical variables significantly associated with ≥F2 fibrosis (Table 2). The discriminatory performance of the clinical model for ≥F2 fibrosis prediction was 0.793 (95% CI 0.657–0.917) with a mean absolute calibration error of 0.290 (95% CI 0.225–0.343). The performance of our clinical model was better than the APRI score (at 3 months post-LT) alone to predict ≥F2 fibrosis (AUC 0.705; 95% CI 0.632–0.777, *p* < 0.001).

**TABLE 2:** Multivariate regression analysis of clinical and radiomics variables.

Statistic/Predictor		Clinical only	Radiomics only	Clinical + radiomics
Mean AUC (95% CI)		0.793 (0.657, 0.917)	0.664 (0.539, 0.775)	0.811 (0.670, 0.921)
Mean Absolute Calibration Error (95% CI)		0.290 (0.225, 0.343)	0.393 (0.320, 0.464)	0.284 (0.221, 0.344)
Venous Original First-Order Maximum			0.52 (0.38, 0.71) *p* < 0.001	0.61 (0.41, 0.92) *p* = 0.019
Primary Diagnosis (ref = Viral)	Autoimmune hepatitis	1.88 (0.52, 6.76) *p* = 0.334		2.15 (0.58, 8.02) *p* = 0.255
	Alcohol	5.49 (1.60, 18.8) *p* = 0.007		4.57 (1.32, 15.90) *p* = 0.018
	NASH	3.12 (0.78, 12.50) *p* = 0.109		2.54 (0.63, 10.20) *p* = 0.191
	Other	2.48 (0.57, 10.83) *p* = 0.228		2.92 (0.65, 13.01) *p* = 0.162
Age at Transplant		0.95 (0.91, 0.98) *p* = 0.004		0.95 (0.92, 0.99) *p* = 0.011
BMI (ref <30)	≥30	1.87 (0.83, 4.22) *p* = 0.134		1.67 (0.73, 3.83) *p* = 0.228
Donor Age		1.04 (1.01, 1.07) *p* = 0.002		1.04 (1.01, 1.06) *p* = 0.006
Post-LT Diabetes (ref = No)	Yes	0.59 (0.29, 1.22) *p* = 0.158		0.60 (0.29, 1.25) *p* = 0.172
Recurrence of Primary Diagnosis (ref = No)	Yes	6.31 (2.46, 16.16) *p* < 0.001		5.01 (1.92, 13.08) *p* = 0.001
Transplant Type (ref = Deceased cardiac donor)	Living donor	0.47 (0.14, 1.55) *p* = 0.214		0.40 (0.12, 1.34) *p* = 0.138
	Deceased brain-dead donor	0.16 (0.05, 0.48) *p* = 0.001		0.15 (0.05, 0.46) *p* = 0.001
Immunosuppressant (ref = Cyclosporine)	Sirolimus	2.05 (0.20, 20.71) *p* = 0.545		1.99 (0.20, 19.62) *p* = 0.555
	Tacrolimus	0.27 (0.11, 0.65) *p* = 0.004		0.27 (0.11, 0.66) *p* = 0.005
Log APRI 3M		1.93 (1.26, 2.95) *p* = 0.003		2.02 (1.31, 3.13) *p* = 0.002

*p*-Values comparing AUC performance.

DeLong’s test was used to compare the AUC, for the following models:

1. Radiomics vs. clinical: *p* < 0.001.

2. Radiomics vs. clinical + radiomics: *p* < 0.001.

3. Clinical vs. clinical + radiomics: *p* = 0.006.

**FIGURE 3:**
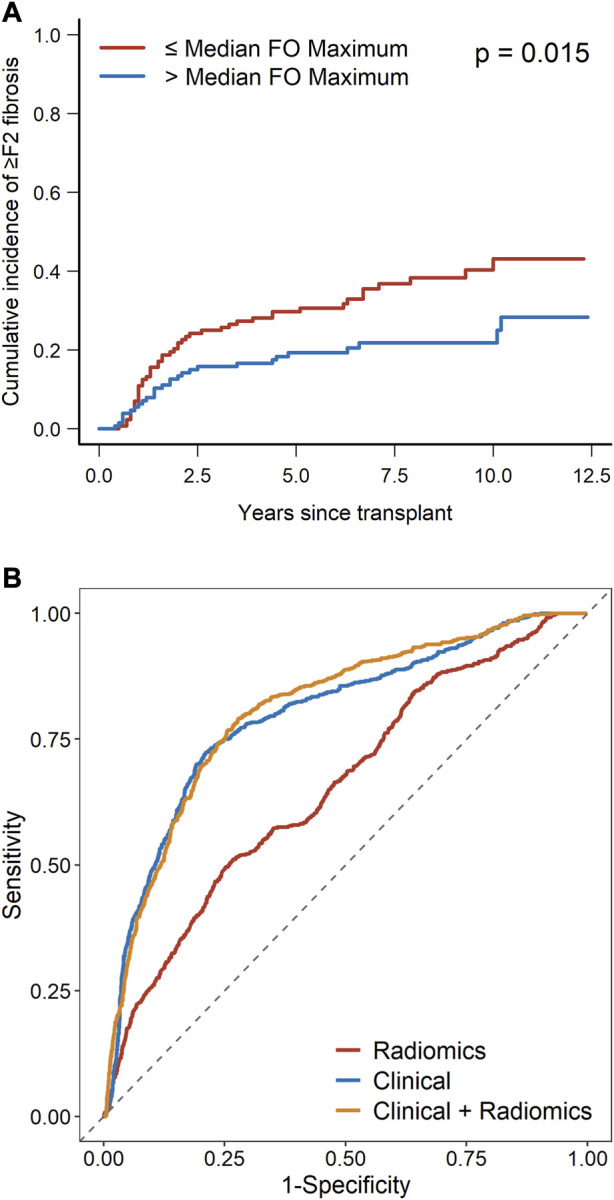
**(A)** Cumulative incidence of ≥F2 fibrosis as stratified by radiomics feature (median venous perfusion original first-order maximum). **(B)** ROC curves for the radiomics model, clinical model, and radiomics plus clinical model.

Among the radiomic features, portal venous phase maximum liver attenuation remains significantly associated with the outcome on multivariate analysis (OR 0.52, 95% CI 0.38–0.71, *p* < 0.001). Using the median value (−0.012) as the cutoff, venous perfusion maximum liver attenuation was significantly associated with a cumulative incidence of ≥F2 fibrosis (*p* = 0.015) as shown in Figure 3A. The combination radiomics and the clinical model increased the AUC to 0.811 (95% CI 0.670–0.921) from 0.793 (95% CI 0.657–0.917) for the clinical-only model (*p* = 0.008) and from 0.664 (95% CI 0.539–0.775) for the radiomics-only model (*p* < 0.001). The mean ROC curves for each model are presented in Figure 3B. Supplementary Figure S2 shows the calibration plots.

Cofounding factor analysis showed a possibility of a small amount of cofounding of radiomics with the primary diagnosis, BMI, recurrence of primary disease, immunosuppression, and type of LT, while no interaction was found with recipient age, donor age, post-LT diabetes, and APRI at 3 months as shown in Table 3 and Supplementary Figure S3.

**TABLE 3:** Univariable and multivariable logistic regression models predicting ≥F2 fibrosis after adjustment for maximum liver attenuation.

Covariate		Unadjusted OR (95% CI)	*p*-value	Adjusted OR (95% CI)	*p*-value
Primary diagnosis (ref = Viral)	AIH	0.83 (0.35, 1.94)	0.66	0.87 (0.36, 2.12)	0.762
	ETOH	0.63 (0.27, 1.44)	0.27	0.51 (0.22, 1.21)	0.129
	NASH	0.76 (0.28, 2.07)	0.59	0.55 (0.19, 1.57)	0.268
	Other	0.58 (0.22, 1.53)	0.27	0.65 (0.23, 1.80)	0.408
	Maximum Liver Attenuation			0.50 (0.37, 0.69)	<0.001
BMI (ref <30)	≥30	1.3 (0.71, 2.39)	0.39	1.00 (0.53, 1.90)	0.999
	Maximum Liver Attenuation			0.52 (0.38, 0.72)	<0.001
Post LT DM (ref = No)	Yes	0.69 (0.4, 1.19)	0.18	0.69 (0.39, 1.22)	0.201
	Maximum Liver Attenuation			0.52 (0.38, 0.71)	<0.001
Recurrent primary disease (ref = No)	Yes	6.23 (3.45, 11.24)	<0.001	5.45 (2.98, 9.98)	<0.001
	Maximum Liver Attenuation			0.58 (0.42, 0.81)	0.0011
Age at Transplant	Age	0.97 (0.95, 1.00)	0.023	0.97 (0.95, 1.00)	0.039
	Maximum Liver Attenuation			0.53 (0.39, 0.72)	<0.001
Donor age	Age	1.01 (0.99, 1.02)	0.45	1.01 (0.99, 1.02)	0.558
	Maximum Liver Attenuation			0.52 (0.38, 0.72)	<0.001
APRI	log APRI 3M	2.12 (1.56, 2.89)	<0.001	2.10 (1.53, 2.87)	<0.001
	Maximum Liver Attenuation			0.51 (0.37, 0.72)	<0.001
Immunosuppressant (ref = Cyclosporine)	Sirolimus	0.50 (0.08, 3.20)	0.47	0.62 (0.09, 4.22)	0.622
	Tacrolimus	0.19 (0.10, 0.34)	<0.001	0.19 (0.10, 0.36)	<0.001
	Maximum Liver Attenuation			0.52 (0.37, 0.73)	<0.001
Transplant type (ref = Deceased cardiac donor)	Living donor	0.59 (0.23, 1.49)	0.27	0.46 (0.17, 1.22)	0.121
	Deceased brain-dead donor	0.31 (0.13, 0.72)	0.007	0.25 (0.10, 0.62)	0.003
	Maximum Liver Attenuation			0.50 (0.37, 0.69)	<0.001

We performed the analysis with biopsy-determined endpoints. In total, 11 patients who had their fibrosis detected using a Fibroscan were excluded from the analysis. Minor differences in model performance were observed. In the radiomics-only, clinical-only, and radiomics + clinical models, the mean AUCs were 0.633, 0.787, and 0.793 for the biopsy-only group as compared to 0.664, 0.793, and 0.811 for the full group, respectively (Supplementary Tables S5, S6).

## Discussion

Radiomics is an emerging but promising imaging-based tool for quantitative analysis of radiological data. Radiomics-based models have been used to detect cirrhosis in the pre-liver transplant setting [14, 27, 28] and have been extensively studied in the cancer setting [29]. In the transplant setting, its application is so far limited to the prediction of recurrent HCC based on pre-transplant images [30]. In a first-of-its-kind study, we evaluated the feasibility of applying radiomic imaging biomarkers in post-transplant CT scans combined with laboratory and clinical data to predict the future development of clinically significant graft fibrosis (Stage 2 or greater) after LT. We appreciate that F4 fibrosis is an important endpoint, however, limiting to F4 only would have dropped the sample size to get a meaningful result. Nonetheless, we believe that identifying patients at risk of developing F2 fibrosis will help us implement measures clinically to prevent its onset.

Radiomic CT data were used to develop a model that would serve to predict graft fibrosis in post-LT patients. The addition of radiomic features to the full clinical model further improved the mean AUC significantly. The maximum liver attenuation value on CT in a representative portion of the right lobe of the liver calculated at the portal venous phase was heavily correlated with the onset of graft fibrosis. As CT enhancement is related to perfusion, greater portal perfusion of the graft may be associated with a lower risk of long-term fibrosis. Previous studies have found that hypoxia, which could arise from low perfusion, is linked to the development of fibrosis [31–33], by upregulating HIF-1α and NF-κB expression, which activates hepatic stellate cells (HSCs), induces epithelial-mesenchymal transition, and increases inflammation. HSCs activation leads to abnormal extracellular matrix deposition, promoting the development of fibrosis. This in turn can lead to vascular resistance, further decreasing the blood flow/liver perfusion. Additionally, activated HSCs also cause sinusoidal vasoconstriction, leading to further hypoxia [31–33]. This negative cycle of events, whereby fibrosis leads to hypoxia which exacerbates fibrosis, suggests the importance of assessing venous perfusion early on to prevent or delay the fibrosis post-transplant.

The analysis of radiomics features was limited in scope to predicting fibrosis. In our exploratory analysis consisting of univariable logistic regression models, we observed that many venous and arterial first-order features were associated with the outcome, specifically, higher values of the feature were associated with decreased odds of fibrosis. However, these features were highly correlated with one another, and therefore only one was selected for the final model to prevent multicollinearity. Beyond these first-order features, no other types of features achieved statistical significance in univariable analysis.

We showed a positive correlation of fibrosis with both the donor’s and recipient’s age, as reported previously in the literature [34, 35]. Increasing donor age was associated with an accelerated rate of fibrosis progression, with a greater fibrosis score both at 4 and 12 months post-transplant [34]. The enhanced fibrotic response observed in older donors could be explained by age-dependent changes in the liver extracellular matrix [35, 36].

Ideally, the model should have included only variables measured closer to the CT scan. However, we anticipated that post-LT diabetes and recurrence of primary disease would have an impact on the incidence of graft fibrosis as supported by the previous literature. Hence these were included in the model. The primary etiology for the transplant and diabetes were among the top 23 ranked features impacting the incidence of graft fibrosis in a recent study based on a deep learning framework [37]. Patients with viral etiology (HBV and HCV) were less likely to develop fibrosis. This could be due to the advent of potent direct-acting antivirals (DAAs) against HBV and HCV in the recent era. This contrasts with the previous literature from the pre-DAA era, which was suggestive of a high rate of fibrosis post-LT in HCV patients [38]. As shown in previous literature, alcohol etiology was related to the highest odds of developing clinically significant fibrosis [39]. We also showed that the recurrence of primary disease was significantly associated with ≥F2 fibrosis post-transplant. In patients with viral infection-related diagnoses, their immunocompromised state post-transplant is further worsened by an increased viral load and an accelerated progression of the disease [34]. Primary sclerosing cholangitis is also known to recur in around 20%–25% of patients over a 10 years period after LT. Given the lack of established treatment, it can rapidly progress leading to graft failure and the need for re-transplantation [40].

The type of LT donor also contributed to the likelihood of developing clinically significant fibrosis post-LT. Recipients from a donor of circulatory death (DCD) were at significantly greater risk of developing severe fibrosis post-LT than those from a neurologically determined dead (NDD) donor or a living donor. Though, an earlier study reported an insignificant difference in fibrosis between DCD and NDD groups [41]. However, the improved prognosis in fibrosis for those with living donors has been previously reported, although mostly with an HCV population, and may be explained by the younger age and shorter cold ischemic times of living donor livers [42, 43].

The immunosuppression regimen was also linked to fibrosis occurrence post-LT, with the use of sirolimus linked to a higher risk for the development of ≥F2 fibrosis and the use of tacrolimus associated with a lower risk when compared to cyclosporin. This was in concordance with previous larger UNOS/SRTR data-based studies showing the superiority of tacrolimus over cyclosporin and sirolimus [44].

While many studies have tested the accuracy of APRI and FIB4 tests in predicting fibrosis in patients with liver diseases, few have investigated their accuracy in the post-LT population [4, 5, 11]. APRI and FIB-4 tests successfully detected fibrosis in post-LT patients with AUCs of 0.87 and 0.78, respectively [45]. In another study, APRI and FIB-4 significantly corresponded with F2 fibrosis on liver biopsy in a post-LT setting (*p* = 0.009 and 0.022, respectively) with sensitivities of 63.4% and 57.7% and specificities of 66.7% and 69.6%, respectively for APRI and Fib-4 [46]. In our cohort, a univariable logistic regression model with APRI at 3 months post-LT obtained an AUC of 0.705 to predict future fibrosis, while a full clinical model, with the removal of correlated variables, returned a mean AUC of 0.803, suggesting the need for a more robust prediction model of fibrosis for post-LT populations.

### Clinical Significance

Recurrent fibrosis following liver transplantation negatively impacts long-term graft and patient survival, increasing the need for re-transplantation. Radiomic features early post-transplant can offer additive prognostic value and insight into the development of significant graft fibrosis in the long term. Due to the lack of correlation between liver enzymes and histology, and the rapid progression of fibrosis in post-transplant patients, there is a need for more robust tools to predict and implement appropriate preventive and therapeutic measures. Based on the current model using clinical and radiomic features, clinicians may consider closer monitoring with Fibroscan in those patients who have high-risk radiomic features and clinically predictive features (therefore higher risk of future F2 fibrosis).

### Limitations

We acknowledge the limitations of the smaller sample size and lack of external validation cohort; however, this was a first-of-its-kind proof of principle study. We also acknowledge the component of ascertainment bias as the number of HCC patients was higher (80%) than usual (40%) in our cohort. This could be due to the retrospective study design and the selection criterion of CT scan done between 3–6 months which is often done for HCC surveillance and not available for non-HCC patients. However, we believe that this would not have impacted the model’s capacity to predict future graft fibrosis as HCC patients were equally distributed in the two groups, and both groups were followed for an equal period. Further, the CT technology changes over the last decade could add some bias. However, limiting the timeframe to more recent dates would reduce the sample size and the follow-up duration. We acknowledge that performing an interobserver variability analysis would have been ideal. However, prior studies have shown that the first-order features found to be significant in this study are amongst the most stable radiomics features with intraclass correlation coefficient (ICC) > 0.9 [47]. Thus, it is reasonable to assume good ICC for this particular radiomics feature. Future work will include further analysis with ICC in particular to assess the usability of second-order features. Moreover, the indications of liver biopsy and other donor factors such as comorbidities, steatosis, liver enzymes, and cold ischemia time were not analyzed, as the major goal of this study was to assess the predictability of radiomic features for graft fibrosis rather than identifying clinical factors affecting graft fibrosis. Furthermore, there was a small amount of confounding for a few clinical variables with radiomic features, hence limiting the increment in AUCs after the addition of radiomics in the clinical model.

## Conclusion

Clinical parameters early post-transplant can prognosticate the future development of clinically significant graft fibrosis. This pilot study exploring the role of radiomics demonstrates that the addition of radiomic features in a clinical model significantly increased the model’s performance. Further studies would be required to investigate the generalizability of this experimental tool.

## Data Availability

The datasets presented in this article are not readily available because the data are not publicly available due to privacy or ethical restrictions. Requests to access the datasets should be directed to the corresponding authors.

## Footnotes

1
https://www.slicer.org/

## Supplementary Material

Supplementary Figure 1 The left panel shows an axial CT image in the portal venous phase with a spherical (green circle) region of interest (ROI) in the periphery of the liver avoiding all blood vessels. The right panels are coronal **(upper right)** and sagittal **(lower right)** reconstructions showing the placement of ROI. The only radiomic feature of interest was the maximum Hounsfield unit value within the ROI in the portal venous phase. The arterial phase radiomic features were not significant.

Supplementary Figure 2 Calibration plots for clinical model and clinical + radiomics model. Dashed lines represent the 95% confidence intervals.

Supplementary Figure 3 Scatterplots **(A–C)** for age at transplant, donor age, APRI score and boxplots **(D–I)** for primary diagnosis, BMI, post LT diabetes, recurrence of primary disease, Transplant type, and immunosuppression type comparing maximum liver attenuation in patients with or without ≥F2 fibrosis.
