# Supplementary material for: Development of a Radiomics-Based Model to Predict Graft Fibrosis in Liver Transplant Recipients: A Pilot Study
Source: Transpl Int. 2023 Sep 1;36:11149. doi: 10.3389/ti.2023.11149 (PMC10503435; doi:10.3389/ti.2023.11149)
Supplement: Supplementary file 3 [file Table1.docx]

**SUPPLEMENTARY MATERIAL**

| **Supplementary Table 1. CT scan acquisition parameters. Arterial phase** | |
| --- | --- |
| **Scan Parameter** | **n = 217** |
| Tube voltage (kVp), n (%) |  |
| 120 | 215 (99.1) |
| 180 | 2 (0.9) |
| Exposure (mAs), Median (min, max) | 67.00 (35.00, 290.00) |
| Exposure Time (sec), Median (min, max) | 500.00 (500.00, 5400.00) |
| Tube Current (mA) , Median (min, max) | 130.00 (50.00, 580.00) |
| Pixel spacing (mm), Median (min, max) | 0.78 (0.54, 2.00) |
| Slice Thickness (mm), n (%) |  |
| 1 | 11 (5.1%) |
| 2 | 1 (0.5%) |
| 3 | 21 (9.7%) |
| 5 | 184 (84.8%) |
| Manufacturer , n (%) |  |
| Toshiba | 217 (100.0%) |
| Manufacturer Model , n (%) |  |
| Aquilion | 190 (87.6%) |
| Aquilion ONE | 22 (10.1%) |
| Aquilion PRIME | 5 (2.3%) |
| Reconstruction Diameter (mm), Median (min, max) | 400.00 (276.56, 1000.00) |
| ConvolutionKernel , n (%) |  |
| FC03 | 9 (4.1%) |
| FC04 | 186 (85.7%) |
| FC07 | 1 (0.5%) |
| FC08 | 3 (1.4%) |
| FC14 | 17 (7.8%) |
| FL03 | 1 (0.5%) |
| Contrast Injection velocity, range, (ml/sec) | 5-7 |
| Time of injection delay (sec) | 15 |
| * Due to the enrolment time, multiple scan parameters had variations during the follow-up period. | |
