# Supplementary material for: Development of a Radiomics-Based Model to Predict Graft Fibrosis in Liver Transplant Recipients: A Pilot Study
Source: Transpl Int. 2023 Sep 1;36:11149. doi: 10.3389/ti.2023.11149 (PMC10503435; doi:10.3389/ti.2023.11149)
Supplement: Supplementary file 4 [file Table2.docx]

| **Supplementary Table 2. CT scan acquisition parameters. Venous phase** | |
| --- | --- |
| **Scan Parameter *** | **n = 254** |
| Tube voltage (kVp), n (%) |  |
| 120 | 253 (99.6) |
| 135 | 1 (0.4) |
| Exposure (mAs), Median (min, max) | 75.00 (40.00, 7000.00) |
| Exposure Time (sec), Median (min, max) | 500.00 (500.00, 1431.00) |
| Tube Current (mA) , Median (min, max) | 150.00 (80.00, 580.00) |
| Pixel spacing (mm), Median (min, max) | 0.78 (0.54, 1.01) |
| Slice Thickness (mm), n (%) |  |
| 1.25 | 1 (0.4) |
| 3 | 28 (11.0) |
| 5 | 225 (88.6) |
| Manufacturer , n (%) |  |
| GE | 2 (0.8) |
| Philips | 1 (0.4) |
| TOSHIBA | 251 (98.8) |
| Manufacturer Model , n (%) |  |
| Aquilion | 215 (84.6) |
| Aquilion ONE | 30 (11.8) |
| Aquilion PRIME | 6 (2.4) |
| iCT 256 | 1 (0.4) |
| LightSpeed QX/i | 1 (0.4) |
| LightSpeed VCT | 1 (0.4) |
| Reconstruction Diameter (mm), Median (min, max) | 400.00 (276.56, 515.62) |
| Convolution Kernel, n(%) |  |
| B | 1 (0.4) |
| FC04 | 222 (87.4) |
| FC08 | 2 (0.8) |
| FC13 | 1 (0.4) |
| FC14 | 25 (9.8) |
| FC17 | 1 (0.4) |
| STANDARD | 2 (0.8) |
| Contrast Injection velocity, range, (ml/sec) | 5-7 |
| Time of injection delay (sec) | 60 |
| * Due to the enrolment time, multiple scan parameters had variations during the follow-up period. | |
