# Supplementary material for: Development of a Radiomics-Based Model to Predict Graft Fibrosis in Liver Transplant Recipients: A Pilot Study
Source: Transpl Int. 2023 Sep 1;36:11149. doi: 10.3389/ti.2023.11149 (PMC10503435; doi:10.3389/ti.2023.11149)
Supplement: Supplementary file 5 [file Table5.docx]

| **Supplementary Table 5: Demographic and clinicopathological characteristics – Excluding Fibroscan determined fibrosis patients** | | | | |
| --- | --- | --- | --- | --- |
| **Variable** | **Full Sample (n=243)** | **<F2 Fibrosis (n=179)** | **≥F2 Fibrosis (n=64)** | **p-value*** |
| Primary diagnosis, n (%) |  |  |  | 0.66 |
| Viral | 131 (54) | 91 (51) | 40 (62) |  |
| Alcohol | 29 (12) | 22 (12) | 7 (11) |  |
| Autoimmune liver diseases | 37 (15) | 29 (16) | 8 (12) |  |
| NASH | 20 (8) | 16 (9) | 4 (6) |  |
| Other | 26 (11) | 21 (12) | 5 (8) |  |
| Liver malignancy pre LT, n (%) |  |  |  | 0.62 |
| Cholangiocarcinoma | 3 (2) | 3 (2) | 0 (0) |  |
| HCC | 188 (96) | 140 (95) | 48 (100) |  |
| HCC + Cholangiocarcinoma | 4 (2) | 4 (3) | 0 (0) |  |
| HCC + Gall bladder carcinoma | 1 (1) | 1 (1) | 0 (0) |  |
| None | 47 | 31 | 16 |  |
| Transplant Type, n (%) |  |  |  | **0.006** |
| Deceased cardiac donor | 24 (10) | 13 (7) | 11 (17) |  |
| Living donor | 60 (25) | 39 (22) | 21 (33) |  |
| Deceased brain dead donor | 159 (65) | 127 (71) | 32 (50) |  |
| Age at transplant (years) |  |  |  | **<0.001** |
| Mean (SD) | 56.3 (10.2) | 57.2 (10.2) | 53.5 (9.9) |  |
| Sex |  |  |  | 0.87 |
| Female | 57 (23) | 41 (23) | 16 (25) |  |
| Male | 186 (77) | 138 (77) | 48 (75) |  |
| BMI (Kg/m^2^) |  |  |  | 0.27 |
| Mean (SD) | 27.0 (5.0) | 27.2 (5.0) | 26.4 (4.9) |  |
| BMI Category |  |  |  | 1 |
| <30 | 184 (76) | 135 (76) | 49 (77) |  |
| >=30 | 58 (24) | 43 (24) | 15 (23) |  |
| Missing | 1 | 1 | 0 |  |
| Donor Age (Years) |  |  |  | 0.96 |
| Mean (SD) | 44.0 (16.6) | 44.0 (17.1) | 43.9 (15.2) |  |
| Diabetes Pre LT | 78 (32) | 58 (32) | 20 (31) | 0.99 |
| Hypertension Pre LT | 80 (33) | 63 (35) | 17 (27) | 0.27 |
| Dyslipidemia Pre LT | 31 (13) | 26 (15) | 5 (8) | 0.41 |
| Cardiovascular disease Pre LT | 19 (8) | 15 (8) | 4 (6) | 0.79 |
| Smoking pre LT | 131 (54) | 98 (55) | 33 (52) | 0.77 |
| Dialysis Pre LT | 2 (1) | 0 (0) | 2 (3) | 0.069 |
| Diabetes Post LT | 120 (49) | 93 (52) | 27 (42) | 0.23 |
| Hypertension Post LT | 145 (60) | 104 (58) | 41 (64) | 0.49 |
| Dyslipidemia Post LT | 66 (27) | 49 (27) | 17 (27) | 1 |
| Cardiovascular disease Post LT | 30 (12) | 21 (12) | 9 (14) | 0.79 |
| Dialysis Post LT | 28 (12) | 19 (11) | 9 (14) | 0.61 |
| Smoking Post LT | 19 (8) | 17 (9) | 2 (3) | 0.17 |
| Alcohol consumption Post LT | 8 (3) | 6 (3) | 2 (3) | 1 |
| HCC/Cholangiocarcinoma Recurrence | 40 (16) | 28 (16) | 12 (19) | 0.7 |
| **Recurrence of the Primary diagnosis** | 85 (35) | 43 (24) | 42 (66) | **<0.001** |
| Platelet at Transplant (x10^9^/L)  Median (Min, Max) | 163 (29,782) | 169 (38,782) | 153 (29,584) | 0.43 |
| Platelets at 3 months (x10^9^/L)  Median (Min, Max) | 158 (15,532) | 162 (39,532) | 148.5 (15.0,446.0) | 0.071 |
| AST at Transplant  Median (Min, Max) | 1042 (96,10300) | 1006 (96,8209) | 1312 (144,10300) | 0.33 |
| **AST** at 3 months (IU/L)  Median (Min, Max) | 28.5 (9.0,358.0) | 26 (9,358) | 44.5 (18.0,268.0) | **<0.001** |
| ALT at Transplant (IU/L)  Median (Min, Max) | 750 (55,7509) | 693 (55,7509) | 854.5 (128.0,5229.0) | 0.41 |
| **ALT** at 3 months (IU/L)  Median (Min, Max) | 36 (3,522) | 30 (3,522) | 63 (9,493) | **<0.001** |
| ALP at Transplant (IU/L)  Median (Min, Max) | 104 (37,1791) | 103 (37,1791) | 106.5 (44.0,1279.0) | 0.78 |
| **ALP** 3 months (IU/L)  Median (Min, Max) | 117 (39,2197) | 108 (39,565) | 131.5 (49.0,2197.0) | **0.005** |
| Total Bilirubin at Transplant (µmol/L)  Median (Min, Max) | 59.5 (6.0,613.0) | 58 (6,613) | 67 (7,512) | 0.16 |
| Total Bilirubin 3 months (µmol/L)  Median (Min, Max) | 10 (3,169) | 9 (3,169) | 13 (4,53) | **<0.001** |
| INR at LT  Median (Min, Max) | 1.8 (0.8,5.2) | 1.8 (0.8,4.0) | 1.8 (1.0,5.2) | 0.71 |
| INR 3 months  Median (Min, Max) | 1.0 (0.9,3.0) | 1.0 (0.9,3.0) | 1.0 (0.9,1.9) | 0.9 |
| Serum Creatinine at Transplant (µmol/L)  Median (Min, Max) | 83 (43,359) | 84 (48,307) | 81 (43,359) | 0.93 |
| Serum Creatinine 3M (µmol/L)  Median (Min, Max) | 90 (28,541) | 91 (28,159) | 86 (44,541) | 0.25 |
| **Serum Sodium** at Transplant (mmol/L)  Mean (SD) | 140.3 (4.4) | 139.9 (4.3) | 141.6 (4.6) | **0.004** |
| Serum Sodium 3 months (mmol/L)  Mean (SD) | 139.9 (3.2) | 139.9 (3.4) | 139.9 (2.7) | 0.91 |
| **Immunosuppressant** 3 months |  |  |  | **<0.001** |
| Cycolosporine | 58 (24) | 27 (15) | 31 (48) |  |
| Sirolimus | 5 (2) | 3 (2) | 2 (3) |  |
| Tacrolimus | 180 (74) | 149 (83) | 31 (48) |  |
| **Notes**: * Mann-Whitney U test for continuous covariates, and Fisher’s Exact test for categorical covariates **Abbreviations**: ALP - Alkaline phosphatase; ALT – Alanine Transaminase; AST – Aspartate Transaminase; BMI – Body mass index; HCC – Hepatocellular carcinoma; INR - International normalized ratio; LT - Liver Transplant; NASH – Non-alcoholic steatohepatitis; SD – Standard deviation | | | | |
