# Supplementary material for: Development of a Radiomics-Based Model to Predict Graft Fibrosis in Liver Transplant Recipients: A Pilot Study
Source: Transpl Int. 2023 Sep 1;36:11149. doi: 10.3389/ti.2023.11149 (PMC10503435; doi:10.3389/ti.2023.11149)
Supplement: Supplementary file 6 [file Table3.docx]

| **Supplementary Table 3. Radiomics parameters (5 mm acquisition)** | | |
| --- | --- | --- |
| **Parameters** | **Definition according PyRadiomics documentation** | **Value** |
| Image Type | Original images were used for feature extraction. No filter (i.e LoG, SquareRoot, etc) were applied. | Original |
| minimumROIDimensions | The minimum dimensions specified. Is an integer in the range 1-3. (1D, 2D or 3D, respectively). | 2 |
| minimumROISize | The minimum number of voxels required to extract the features. | None |
| Normalize | Is Set to False to disable image normalisation prior to resampling. | False |
| normalizeScale | Scale for image normalization. This has no effect if normalising is disabled. | 1 |
| removeOutliers | The outliers to remove from the image. If this parameter is omitted, no outliers are removed. | None |
| resampledPixelSpacing | This list of three floats (>= 0) determines the size of the resampling voxel in the (x, y, z) plane. | [5_5_5] |
| Interpolator | Sets the interpolator to use for resampling using. The interpolator is only used when resampling images. sitkBSpline is equal to the integer 3. | sitkBSpline |
| preCrop | If true and resampling is disabled, this crops image to the bounding box with additional padding specified by padDistance. | False |
| padDistance | Set the number of voxels to pad cropped volume with during resampling. Only works when preCrop is enabled. | 10 |
| Distances | Distances between the centre voxel and its neighbour for which angles should be generated. | 1 |
| force2D | Set to true to force a texture calculation by slice. | False |
| force2Ddimension | Specifies the 'slice' dimension for a by-slice feature extraction. | 0 |
| resegmentRange | Specifies the lower and optionally upper thresholds prior to feature calculation, segmented voxels outside of this range are removed. | None |
| Label | A label value of Region of Interest (ROI) in labelmap. | 1 |
| additionalInfo | Set to False to disable the inclusion of additional information on the extraction in the output. | True |
| binWidth | It is the size of the bins used to create a histogram and to discretize the grey level of an image. | 25 |
| A more complete definition of parameter settings can be found in the online PyRadiomics documentation (1)   1. Customizing the Extraction — pyradiomics v3.0.post5+gf06ac1d documentation [Internet]. [cited 2021 Jul 27] Available from: https://pyradiomics.readthedocs.io/en/latest/customization.html | | |
