# Supplementary material for: Development of a Radiomics-Based Model to Predict Graft Fibrosis in Liver Transplant Recipients: A Pilot Study
Source: Transpl Int. 2023 Sep 1;36:11149. doi: 10.3389/ti.2023.11149 (PMC10503435; doi:10.3389/ti.2023.11149)
Supplement: Supplementary file 7 [file Table4.docx]

| **Supplementary Table 4: Univariable Logistic Regression Models for Radiomics Features** | | | |
| --- | --- | --- | --- |
| **Feature** | **OR** | **95% CI** | **p-value** |
| venous original shape Elongation | 1.11 | (0.83, 1.46) | 0.48 |
| venous original shape Flatness | 1.13 | (0.86, 1.49) | 0.36 |
| venous original shape Least Axis Length | 0.95 | (0.72, 1.27) | 0.74 |
| venous original shape Major Axis Length | 0.80 | (0.50, 1.30) | 0.37 |
| venous original shape Maximum2DDiameterColumn | 1.00 | (0.76, 1.31) | 1 |
| venous original shape Maximum2DDiameterRow | 0.82 | (0.59, 1.15) | 0.25 |
| venous original shape Maximum2DDiameterSlice | 0.82 | (0.57, 1.17) | 0.28 |
| venous original shape Maximum3DDiameter | 0.84 | (0.56, 1.25) | 0.39 |
| venous original shape Mesh Volume | 0.95 | (0.71, 1.27) | 0.72 |
| venous original shape Minor Axis Length | 0.92 | (0.68, 1.23) | 0.56 |
| venous original shape Sphericity | 1.15 | (0.87, 1.52) | 0.34 |
| venous original shape Surface Area | 0.92 | (0.68, 1.24) | 0.58 |
| venous original shape Surface Volume Ratio | 1.09 | (0.81, 1.47) | 0.56 |
| venous original shape Voxel Volume | 0.95 | (0.71, 1.27) | 0.71 |
| **venous original first order 10Percentile** | 0.53 | (0.39, 0.72) | **<0.001** |
| **venous original first order 90Percentile** | 0.51 | (0.37, 0.69) | **<0.001** |
| **venous original first order Energy** | 0.51 | (0.28, 0.91) | **0.022** |
| venous original first order Entropy | 0.94 | (0.72, 1.24) | 0.67 |
| venous original first order Interquartile Range | 0.78 | (0.55, 1.09) | 0.15 |
| venous original first order Kurtosis | 1.11 | (0.86, 1.44) | 0.41 |
| **venous original first order Maximum** | 0.52 | (0.38, 0.71) | **<0.001** |
| venous original first order Mean Absolute Deviation | 0.81 | (0.58, 1.14) | 0.23 |
| **venous original first order Mean** | 0.50 | (0.37, 0.68) | **<0.001** |
| **venous original first order Median** | 0.50 | (0.37, 0.69) | **<0.001** |
| **venous original first order Minimum** | 0.58 | (0.43, 0.78) | **<0.001** |
| venous original first order Range | 0.87 | (0.65, 1.16) | 0.34 |
| venous original first order Robust Mean Absolute Deviation | 0.81 | (0.57, 1.15) | 0.23 |
| **venous original first order Root Mean Squared** | 0.50 | (0.36, 0.68) | **<0.001** |
| venous original first order Skewness | 0.89 | (0.68, 1.16) | 0.38 |
| **venous original first order Total Energy** | 0.51 | (0.28, 0.91) | **0.022** |
| venous original first order Uniformity | 1.06 | (0.81, 1.39) | 0.67 |
| venous original first order Variance | 0.76 | (0.45, 1.28) | 0.3 |
| venous original glcm Autocorrelation | 1.05 | (0.81, 1.36) | 0.73 |
| venous original glcm Cluster Prominence | 0.54 | (0.03, 10.73) | 0.69 |
| venous original glcm Cluster Shade | 1.08 | (0.74, 1.56) | 0.7 |
| venous original glcm Cluster Tendency | 0.80 | (0.45, 1.43) | 0.46 |
| venous original glcm Contrast | 0.90 | (0.64, 1.26) | 0.54 |
| venous original glcm Correlation | 0.88 | (0.67, 1.17) | 0.38 |
| venous original glcm Difference Average | 0.95 | (0.72, 1.25) | 0.7 |
| venous original glcm Difference Entropy | 0.92 | (0.70, 1.21) | 0.57 |
| venous original glcm Difference Variance | 0.89 | (0.63, 1.26) | 0.5 |
| venous original glcm Id | 1.02 | (0.78, 1.33) | 0.91 |
| venous original glcm Idm | 1.02 | (0.78, 1.34) | 0.86 |
| venous original glcm Idmn | 1.06 | (0.81, 1.39) | 0.68 |
| venous original glcm Idn | 1.03 | (0.79, 1.35) | 0.84 |
| venous original glcm Imc1 | 0.93 | (0.71, 1.21) | 0.57 |
| venous original glcm Imc2 | 1.10 | (0.84, 1.45) | 0.48 |
| venous original glcm Inverse Variance | 1.12 | (0.84, 1.48) | 0.45 |
| venous original glcm Joint Average | 1.08 | (0.83, 1.41) | 0.57 |
| venous original glcm Joint Energy | 1.00 | (0.76, 1.31) | 1 |
| venous original glcm Joint Entropy | 0.96 | (0.73, 1.26) | 0.76 |
| venous original glcm MCC | 1.09 | (0.83, 1.42) | 0.54 |
| venous original glcm Maximum Probability | 0.99 | (0.76, 1.30) | 0.94 |
| venous original glcm Sum Average | 1.08 | (0.83, 1.41) | 0.57 |
| venous original glcm Sum Entropy | 0.92 | (0.70, 1.21) | 0.54 |
| venous original glcm Sum Squares | 0.86 | (0.56, 1.32) | 0.48 |
| venous original glrlm Gray Level Non-Uniformity | 0.97 | (0.73, 1.28) | 0.83 |
| venous original glrlm Gray Level Non-Uniformity Normalized | 1.08 | (0.83, 1.42) | 0.56 |
| venous original glrlm Gray Level Variance | 0.87 | (0.59, 1.28) | 0.47 |
| venous original glrlm High Gray Level Run Emphasis | 1.03 | (0.79, 1.34) | 0.81 |
| venous original glrlm Long Run Emphasis | 0.94 | (0.71, 1.23) | 0.65 |
| venous original glrlm Long Run High Gray Level Emphasis | 1.06 | (0.81, 1.37) | 0.69 |
| venous original glrlm Long Run Low Gray Level Emphasis | 0.84 | (0.63, 1.12) | 0.24 |
| venous original glrlm Low Gray Level Run Emphasis | 0.85 | (0.65, 1.13) | 0.27 |
| venous original glrlm Run Entropy | 0.87 | (0.66, 1.15) | 0.32 |
| venous original glrlm Run Length Non-Uniformity | 0.95 | (0.71, 1.27) | 0.73 |
| venous original glrlm Run Length Non-Uniformity Normalized | 1.02 | (0.78, 1.33) | 0.91 |
| venous original glrlm Run Percentage | 1.03 | (0.79, 1.35) | 0.81 |
| venous original glrlm Run Variance | 0.90 | (0.68, 1.19) | 0.46 |
| venous original glrlm Short Run Emphasis | 1.01 | (0.77, 1.32) | 0.95 |
| venous original glrlm Short Run High Gray Level Emphasis | 1.02 | (0.78, 1.33) | 0.87 |
| venous original glrlm Short Run Low Gray Level Emphasis | 0.86 | (0.65, 1.13) | 0.28 |
| venous original glszm Gray Level Non-Uniformity | 0.92 | (0.69, 1.22) | 0.55 |
| venous original glszm Gray Level Non-Uniformity Normalized | 1.00 | (0.77, 1.31) | 0.97 |
| venous original glszm Gray Level Variance | 1.02 | (0.79, 1.34) | 0.86 |
| venous original glszm High Gray Level Zone Emphasis | 0.95 | (0.72, 1.25) | 0.7 |
| venous original glszm Large Area Emphasis | 0.98 | (0.74, 1.29) | 0.87 |
| venous original glszm Large Area High Gray Level Emphasis | 1.02 | (0.78, 1.35) | 0.87 |
| venous original glszm Large Area Low Gray Level Emphasis | 0.88 | (0.64, 1.20) | 0.42 |
| venous original glszm Low Gray Level Zone Emphasis | 1.18 | (0.90, 1.54) | 0.24 |
| venous original glszm Size Zone Non-Uniformity | 0.87 | (0.64, 1.17) | 0.35 |
| venous original glszm Size Zone Non-Uniformity Normalized | 0.94 | (0.71, 1.23) | 0.64 |
| venous original glszm Small Area Emphasis | 1.01 | (0.77, 1.32) | 0.95 |
| venous original glszm Small Area High Gray Level Emphasis | 0.92 | (0.70, 1.21) | 0.54 |
| venous original glszm Small Area Low Gray Level Emphasis | 1.19 | (0.91, 1.55) | 0.21 |
| venous original glszm Zone Entropy | 1.00 | (0.77, 1.31) | 0.98 |
| venous original glszm Zone Percentage | 0.97 | (0.74, 1.28) | 0.82 |
| venous original glszm Zone Variance | 1.00 | (0.76, 1.31) | 0.98 |
| venous original gldm Dependence Entropy | 0.87 | (0.66, 1.16) | 0.36 |
| venous original gldm Dependence Non-Uniformity | 0.97 | (0.73, 1.29) | 0.84 |
| venous original gldm Dependence Non-Uniformity Normalized | 1.08 | (0.83, 1.41) | 0.56 |
| venous original gldm Dependence Variance | 0.89 | (0.67, 1.17) | 0.39 |
| venous original gldm Gray Level Non-Uniformity | 0.96 | (0.72, 1.27) | 0.76 |
| venous original gldm Gray Level Variance | 0.87 | (0.58, 1.29) | 0.48 |
| venous original gldm High Gray Level Emphasis | 1.04 | (0.80, 1.35) | 0.78 |
| venous original gldm Large Dependence Emphasis | 0.93 | (0.71, 1.23) | 0.62 |
| venous original gldm Large Dependence High Gray Level Emphasis | 1.05 | (0.81, 1.37) | 0.71 |
| venous original gldm Large Dependence Low Gray Level Emphasis | 0.85 | (0.63, 1.14) | 0.28 |
| venous original gldm Low Gray Level Emphasis | 0.84 | (0.63, 1.11) | 0.23 |
| venous original gldm Small Dependence Emphasis | 0.94 | (0.72, 1.24) | 0.68 |
| venous original gldm Small Dependence High Gray Level Emphasis | 0.86 | (0.61, 1.22) | 0.41 |
| venous original gldm Small Dependence Low Gray Level Emphasis | 1.14 | (0.88, 1.49) | 0.32 |
| venous original ngtdm Busyness | 0.30 | (0.03, 3.36) | 0.33 |
| venous original ngtdm Coarseness | 1.02 | (0.78, 1.33) | 0.89 |
| venous original ngtdm Complexity | 0.92 | (0.66, 1.28) | 0.61 |
| venous original ngtdm Contrast | 0.91 | (0.69, 1.21) | 0.53 |
| venous original ngtdm Strength | 0.97 | (0.74, 1.28) | 0.85 |
| arterial original shape Elongation | 1.08 | (0.77, 1.50) | 0.66 |
| arterial original shape Flatness | 1.00 | (0.74, 1.35) | 1 |
| arterial original shape Least Axis Length | 0.99 | (0.72, 1.36) | 0.93 |
| arterial original shape Major Axis Length | 0.76 | (0.29, 2.02) | 0.58 |
| arterial original shape Maximum 2D Diameter Column | 1.00 | (0.73, 1.36) | 0.99 |
| arterial original shape Maximum 2D Diameter Row | 0.98 | (0.71, 1.34) | 0.89 |
| arterial original shape Maximum 2D Diameter Slice | 0.89 | (0.61, 1.30) | 0.55 |
| arterial original shape Maximum 3D Diameter | 0.80 | (0.42, 1.53) | 0.5 |
| arterial original shape Mesh Volume | 1.02 | (0.74, 1.40) | 0.9 |
| arterial original shape Minor Axis Length | 1.02 | (0.75, 1.40) | 0.88 |
| arterial original shape Sphericity | 1.18 | (0.82, 1.71) | 0.37 |
| arterial original shape Surface Area | 1.00 | (0.72, 1.37) | 0.98 |
| arterial original shape Surface Volume Ratio | 0.95 | (0.70, 1.28) | 0.72 |
| arterial original shape Voxel Volume | 1.02 | (0.74, 1.40) | 0.92 |
| arterial original first order 10 Percentile | 1.08 | (0.76, 1.54) | 0.66 |
| **arterial original first order 90 Percentile** | 0.64 | (0.46, 0.89) | **0.0086** |
| **arterial original first order Energy** | 0.00 | 2e-06, 0.40) | **0.023** |
| arterial original first order Entropy | 0.91 | (0.66, 1.24) | 0.54 |
| arterial original first order Interquartile Range | 0.80 | (0.44, 1.44) | 0.45 |
| arterial original first order Kurtosis | 0.93 | (0.66, 1.30) | 0.67 |
| **arterial original first order Maximum** | 0.69 | (0.50, 0.96) | **0.027** |
| arterial original first order Mean Absolute Deviation | 0.74 | (0.36, 1.53) | 0.42 |
| arterial original first order Mean | 0.96 | (0.72, 1.27) | 0.75 |
| **arterial original first order Median** | 0.65 | (0.47, 0.90) | **0.01** |
| arterial original first order Minimum | 1.15 | (0.78, 1.71) | 0.48 |
| arterial original first order Range | 0.72 | (0.38, 1.39) | 0.33 |
| arterial original first order Robust Mean Absolute Deviation | 0.78 | (0.42, 1.47) | 0.45 |
| **arterial original first order Root Mean Squared** | 0.24 | (0.09, 0.62) | **0.0033** |
| arterial original first order Skewness | 1.04 | (0.77, 1.41) | 0.81 |
| **arterial original first order Total Energy** | 0.00 | (4.2e-06, 0.40) | **0.023** |
| arterial original first order Uniformity | 1.08 | (0.80, 1.46) | 0.63 |
| arterial original first order Variance | 0.54 | (0.06, 4.83) | 0.58 |
| arterial original glcm Auto correlation | 0.50 | (0.04, 5.69) | 0.58 |
| arterial original glcm Cluster Prominence | 0.02 | (6.3e-12, 4.9e+07) | 0.72 |
| arterial original glcm Cluster Shade | 27.60 | (3.6e-27, 2.1e+29) | 0.92 |
| arterial original glcm Cluster Tendency | 0.44 | (0.02, 8.91) | 0.59 |
| arterial original glcm Contrast | 0.69 | (0.19, 2.51) | 0.57 |
| arterial original glcm Correlation | 1.10 | (0.83, 1.45) | 0.52 |
| arterial original glcm Difference Average | 0.82 | (0.48, 1.40) | 0.47 |
| arterial original glcm Difference Entropy | 0.89 | (0.64, 1.22) | 0.47 |
| arterial original glcm Difference Variance | 0.53 | (0.06, 4.28) | 0.55 |
| arterial original glcm Id | 1.14 | (0.83, 1.56) | 0.41 |
| arterial original glcm Idm | 1.14 | (0.83, 1.55) | 0.42 |
| arterial original glcm Idmn | 0.98 | (0.73, 1.33) | 0.91 |
| arterial original glcm Idn | 1.01 | (0.75, 1.36) | 0.95 |
| arterial original glcm Imc1 | 1.04 | (0.76, 1.41) | 0.82 |
| arterial original glcm Imc2 | 0.95 | (0.70, 1.28) | 0.73 |
| arterial original glcm Inverse Variance | 0.93 | (0.69, 1.25) | 0.62 |
| arterial original glcm Joint Average | 0.68 | (0.31, 1.48) | 0.33 |
| arterial original glcm Joint Energy | 1.06 | (0.79, 1.43) | 0.71 |
| arterial original glcm Joint Entropy | 0.92 | (0.68, 1.25) | 0.6 |
| arterial original glcm MCC | 0.93 | (0.68, 1.26) | 0.64 |
| arterial original glcm Maximum Probability | 1.08 | (0.80, 1.45) | 0.64 |
| arterial original glcm Sum Average | 0.68 | (0.31, 1.48) | 0.33 |
| arterial original glcm Sum Entropy | 0.95 | (0.70, 1.29) | 0.74 |
| arterial original glcm Sum Squares | 0.56 | (0.08, 3.96) | 0.56 |
| arterial original glrlm Gray Level Non-Uniformity | 1.06 | (0.78, 1.44) | 0.71 |
| arterial original glrlm Gray Level Non-Uniformity Normalized | 1.08 | (0.80, 1.46) | 0.62 |
| arterial original glrlm Gray Level Variance | 0.52 | (0.05, 5.15) | 0.58 |
| arterial original glrlm High Gray Level Run Emphasis | 0.53 | (0.07, 3.82) | 0.53 |
| arterial original glrlm Long Run Emphasis | 1.12 | (0.83, 1.52) | 0.45 |
| arterial original glrlm Long Run High Gray Level Emphasis | 0.50 | (0.06, 4.35) | 0.53 |
| arterial original glrlm Long Run Low Gray Level Emphasis | 1.05 | (0.78, 1.41) | 0.73 |
| arterial original glrlm Low Gray Level Run Emphasis | 1.07 | (0.80, 1.44) | 0.64 |
| arterial original glrlm Run Entropy | 0.94 | (0.69, 1.28) | 0.69 |
| arterial original glrlm Run Length Non-Uniformity | 0.95 | (0.68, 1.33) | 0.77 |
| arterial original glrlm Run Length Non-Uniformity Normalized | 0.88 | (0.65, 1.20) | 0.42 |
| arterial original glrlm Run Percentage | 0.88 | (0.65, 1.19) | 0.4 |
| arterial original glrlm Run Variance | 1.10 | (0.81, 1.48) | 0.55 |
| arterial original glrlm Short Run Emphasis | 0.88 | (0.65, 1.18) | 0.39 |
| arterial original glrlm ShortRun High Gray Level Emphasis | 0.53 | (0.07, 4.00) | 0.54 |
| arterial original glrlm Short Run Low Gray Level Emphasis | 1.09 | (0.81, 1.46) | 0.58 |
| arterial original glszm Gray Level Non-Uniformity | 1.01 | (0.75, 1.36) | 0.94 |
| arterial original glszm Gray Level Non-Uniformity Normalized | 1.00 | (0.74, 1.34) | 0.99 |
| arterial original glszm Gray Level Variance | 0.58 | (0.13, 2.64) | 0.48 |
| arterial original glszm High Gray Level Zone Emphasis | 0.42 | 0.01, 13.90) | 0.63 |
| arterial original glszm Large Area Emphasis | 1.06 | (0.78, 1.43) | 0.72 |
| arterial original glszm Large Area High Gray Level Emphasis | 0.13 | (1.9e-04, 92.61) | 0.54 |
| arterial original glszm Large Area Low Gray Level Emphasis | 1.03 | (0.77, 1.38) | 0.83 |
| arterial original glszm Low Gray Level Zone Emphasis | 1.26 | (0.94, 1.70) | 0.13 |
| arterial original glszm Size Zone Non-Uniformity | 0.90 | (0.64, 1.26) | 0.53 |
| arterial original glszm Size Zone Non-Uniformity Normalized | 0.98 | (0.72, 1.33) | 0.9 |
| arterial original glszm Small Area Emphasis | 0.93 | (0.69, 1.26) | 0.66 |
| arterial original glszm Small Area High Gray Level Emphasis | 0.18 | (9.3e-04, 34.63) | 0.52 |
| arterial original glszm Small Area Low Gray Level Emphasis | 1.16 | (0.86, 1.56) | 0.32 |
| arterial original glszm Zone Entropy | 0.91 | (0.67, 1.23) | 0.53 |
| arterial original glszm Zone Percentage | 0.93 | (0.68, 1.27) | 0.65 |
| arterial original glszm Zone Variance | 1.06 | (0.78, 1.45) | 0.71 |
| arterial original gldm Dependence Entropy | 1.02 | (0.76, 1.39) | 0.88 |
| arterial original gldm Dependence Non-Uniformity | 0.99 | (0.72, 1.34) | 0.93 |
| arterial original gldm Dependence Non-Uniformity Normalized | 0.95 | (0.70, 1.29) | 0.76 |
| arterial original gldm Dependence Variance | 1.04 | (0.77, 1.41) | 0.8 |
| arterial original gldm Gray Level Non-Uniformity | 1.07 | (0.78, 1.45) | 0.68 |
| arterial original gldm Gray Level Variance | 0.53 | (0.05, 5.09) | 0.58 |
| arterial original gldm High Gray Level Emphasis | 0.54 | (0.08, 3.76) | 0.53 |
| arterial original gldm Large Dependence Emphasis | 1.11 | (0.82, 1.49) | 0.51 |
| arterial original gldm Large Dependence High Gray Level Emphasis | 0.33 | (0.01, 9.77) | 0.52 |
| arterial original gldm Large Dependence Low Gray Level Emphasis | 1.01 | (0.75, 1.36) | 0.92 |
| arterial original gldm Low Gray Level Emphasis | 1.06 | (0.79, 1.42) | 0.72 |
| arterial original gldm Small Dependence Emphasis | 0.88 | (0.64, 1.20) | 0.41 |
| arterial original gldm Small Dependence High Gray Level Emphasis | 0.42 | (0.03, 6.94) | 0.55 |
| arterial original gldm Small Dependence Low Gray Level Emphasis | 1.09 | (0.81, 1.46) | 0.56 |
| arterial original ngtdm Busyness | 1.02 | (0.77, 1.36) | 0.89 |
| arterial original ngtdm Coarseness | 0.99 | (0.73, 1.34) | 0.95 |
| arterial original ngtdm Complexity | 0.53 | (0.06, 4.80) | 0.57 |
| arterial original ngtdm Contrast | 0.61 | (0.13, 2.89) | 0.53 |
| arterial original ngtdm Strength | 0.53 | (0.11, 2.68) | 0.44 |
