# Supplementary material for: Development of a Radiomics-Based Model to Predict Graft Fibrosis in Liver Transplant Recipients: A Pilot Study
Source: Transpl Int. 2023 Sep 1;36:11149. doi: 10.3389/ti.2023.11149 (PMC10503435; doi:10.3389/ti.2023.11149)
Supplement: Supplementary file 8 [file Table6.docx]

| **Supplementary Table 6: Multivariate regression analysis of clinical and radiomics variables - Excluding Fibroscan determined fibrosis patients.** | | | | |
| --- | --- | --- | --- | --- |
| **Statistic/Predictor** | | **Clinical Only** | **Radiomics Only** | **Clinical + Radiomics** |
| Mean AUC  (95% CI) | | 0.787 | 0.633 | 0.793 |
| Venous Original First-Order Maximum | |  | 0.57  (0.41, 0.78)  p<0.001 | 0.64   (0.42, 0.98)   p=0.043 |
| Primary Diagnosis  (ref=Viral) | Autoimmune hepatitis | 1.49  (0.38, 5.84) p=0.57 |  | - 1. (0.41, 6.74)   2. p=0.473 |
|  | Alcohol | 4.98  (1.4, 17.8) p=0.014 |  | 4.15  (1.15, 15.04)  p=0.031 |
|  | NASH | 2.94  (0.64, 13.56) p=0.169 |  | 2.27  (0.49, 10.53)  p=0.297 |
|  | Other | 2.17  (0.45, 10.38) p=0.335 |  | 2.44  (0.5, 11.89)  p=0.272 |
| Age at Transplant |  | 0.94  (0.91, 0.98) p=0.004 |  | 0.95  (0.91, 0.99)  p=0.007 |
| BMI  (ref = ≤30) | >30 | 1.21  (0.5, 2.93) p=0.678 |  | 1.1  (0.44, 2.71)  p=0.843 |
| Donor Age |  | 1.03  (1.01, 1.06) p=0.014 |  | 1.03  (1, 1.06)  p=0.026 |
| Post-LT Diabetes (ref=No) | Yes | 0.59  (0.28, 1.24) p=0.166 |  | 0.6  (0.28, 1.28)  p=0.184 |
| Recurrence of Primary Diagnosis  (ref=No) | Yes | 4.92  (1.84, 13.17) p=0.002 |  | 3.92  (1.44, 10.67)  p=0.008 |
| Transplant Type  (ref=Deceased cardiac donor) | Living donor | 0.51  (0.15, 1.81) p=0.301 |  | 0.95  (0.91, 0.99)  p=0.008 |
|  | Deceased brain-dead donor | 0.19  (0.06, 0.61) p=0.006 |  | (0.44, 2.71)  p=0.844 |
| Immunosuppressant  (ref=Cyclosporine) | Sirolimus | 2.02  (0.20, 20.62) p=0.554 |  | 2.07  (0.20, 21.50)  p=0.542 |
|  | Tacrolimus | 0.26  (0.10, 0.68) p=0.006 |  | 0.27  (0.11, 0.69)  p=0.006 |
| Log APRI 3M |  | 2.16  (1.39, 3.35) p=0.001 |  | 3.92  (1.44, 10.67)  p=0.009 |
